# Supplementary material for: Quadrupolar excitons and hybridized interlayer Mott insulator in a trilayer moiré superlattice
Source: Nat Commun. 2023 Aug 1;14:4604. doi: 10.1038/s41467-023-40288-9 (PMC10393975; doi:10.1038/s41467-023-40288-9)
Supplement: Supplementary file 1 — Supplementary Information [file 41467_2023_40288_MOESM1_ESM.pdf]

Supplementary Information

Quadrupolar Excitons and Hybridized Interlayer Mott Insulator in a Trilayer Moiré Superlattice

Zhen Lian[1#], Dongxue Chen[1#], Lei Ma[1#], Yuze Meng[1#], Ying Su[2], Li Yan[1], Xiong Huang[3,4], Qiran Wu[3], Xinyue Chen[1], Mark Blei[5], Takashi Taniguchi[6], Kenji Watanabe[7], Sefaattin Tongay[5], Chuanwei Zhang[2], Yong-Tao Cui[3*], Su-Fei Shi[1,8*]

1. Department of Chemical and Biological Engineering, Rensselaer Polytechnic Institute, Troy, NY 12180, USA
2. Department of Physics, University of Texas at Dallas, Dallas, Texas, 75083, USA
3. Department of Physics and Astronomy, University of California, Riverside, California, 92521, USA
4. Department of Materials Science and Engineering, University of California, Riverside, California, 92521, USA
5. School for Engineering of Matter, Transport and Energy, Arizona State University, Tempe, AZ 85287, USA
6. International Center for Materials Nanoarchitectonics, National Institute for Materials Science, 1-1 Namiki, Tsukuba 305-0044, Japan
7. Research Center for Functional Materials, National Institute for Materials Science, 1-1 Namiki, Tsukuba 305-0044, Japan
8. Department of Electrical, Computer & Systems Engineering, Rensselaer Polytechnic Institute, Troy, NY 12180, USA

[#] These authors contributed equally to this work
[*] Corresponding authors: yongtao.cui@ucr.edu, shis2@rpi.edu

Supplementary section 1: Optical microscope image of device D5 and D1

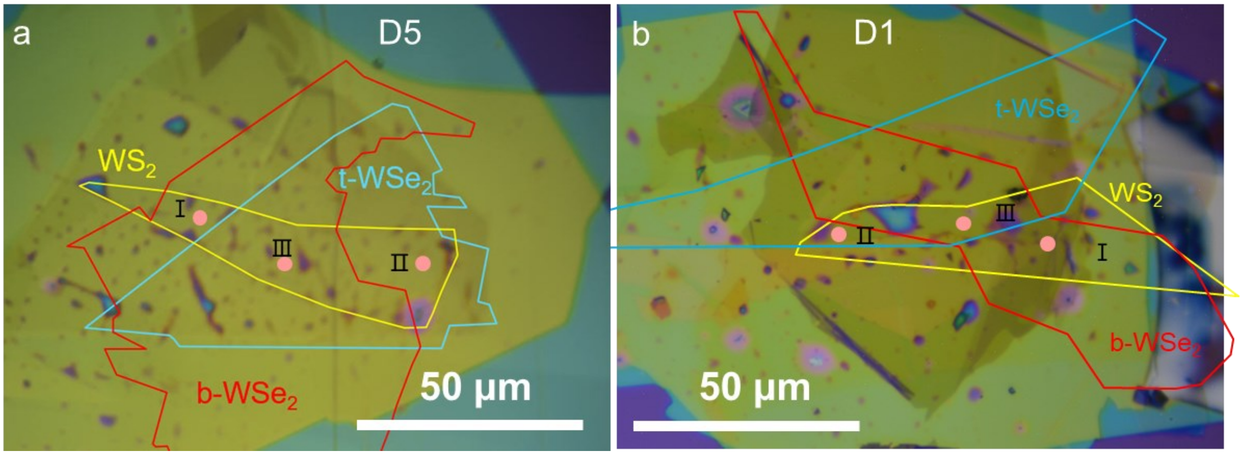

Figure S1. Optical microscope images of the devices shown in the main text. (a) and (b) are the optical images of device D5 and D1, respectively. The pink dots indicate the laser excitation spots for optical measurements.

Supplementary section 2: SHG measurement and determination of alignment angles

Figure S2a and c show normalized SHG intensities as functions of the half-waveplate angle measured on different regions of device D5 and D1. The orientation of the armchair direction of each layer can be obtained by fitting the data with a sinusoidal function.

For device D5, we obtained 1.4° ± 0.5° alignment between b-WSe_2 and WS_2 and -0.1°± 0.5° alignment between t-WSe_2 and WS_2.

For device D1, we obtained 0.9° ± 0.5° alignment between b-WSe_2 and region I and 1.2° ± 0.5° alignment between t-WSe_2 and region I.

The SHG intensity from region I and region II (t-WSe_2/WS_2) are enhanced compared to the SHG intensities from both WSe_2 layers, indicating a close to 0° alignment for both heterobilayers (Figure S2b and d) in D5 and D1.

The lattice constant of WSe_2 and WS_2, a_{Se} and a_S were chosen to 0.328 nm and 0.315 nm. The moiré lattice constant can be calculated by $a_m = \frac{4\pi}{\sqrt{3}|\boldsymbol{k}_{Se} - \boldsymbol{k}_S|}$, where $\boldsymbol{k}_{Se}$ and $\boldsymbol{k}_S$ are reciprocal primitive vectors of WSe_2 and WS_2, respectively. The moiré density n_0 is given by $\frac{2}{\sqrt{3}a_m^2}$. Using the alignment angles determined by SHG, we estimate $n_0 = 2.1 \times 10^{12} cm^{-2}$ for region I and $n_0 = 2.3 \times 10^{12} cm^{-2}$ for region II for device D1 shown in Figs.3 and 4.

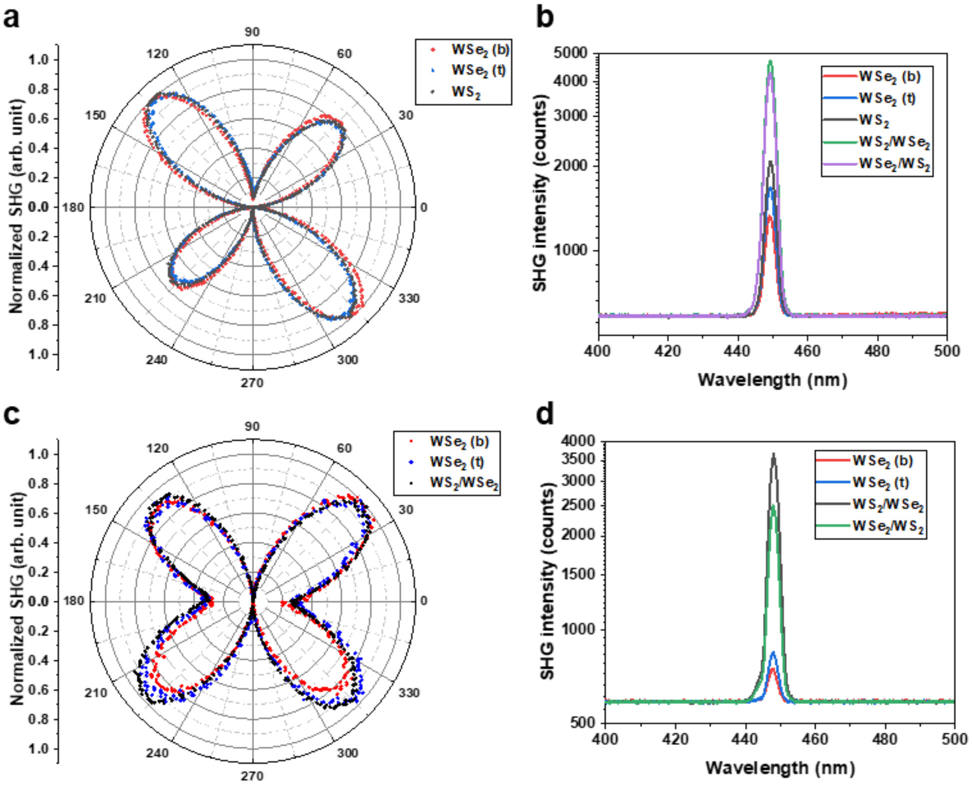

Figure S2. Polarized SHG measurement from different regions of device D5 and D1 shown in main text. (a) shows the SHG intensity as a function of half-waveplate angle measured on b-WSe_2, t-WSe_2 and WS_2 of device D5. (b) shows the relative SHG intensity from b-WSe_2, t-WSe_2, WS_2, region I (WS_2/ WSe_2) and region II (WSe_2/ WS_2) of device D5. (c) shows the SHG intensity as a function of half-waveplate angle measured on b-WSe_2, t-WSe_2 and region I (WS_2/WSe_2) of device D1. (d) shows the relative SHG intensity from b-WSe_2, t-WSe_2, region I (WS_2/ WSe_2) and region II (WSe_2/ WS_2) of device D1. The enhancement of SHG signal on region I and region II indicates the top two layers and the bottom two layers are both close-to-zero-degree-aligned in both devices.

Supplementary section 3: Comparison of the PL spectra at zero gate voltage in the bilayer regions and the trilayer region

Figure S3 shows the PL spectra from region I (WS_2/b-WSe_2), region II (t-WSe_2/WS_2) and region III (t-WSe_2/WS_2/b-WSe_2) of device D5 at zero gate voltage. All the PL spectra were taken at 6 K using 50 uW 633 nm excitation and the same integration time (2s).

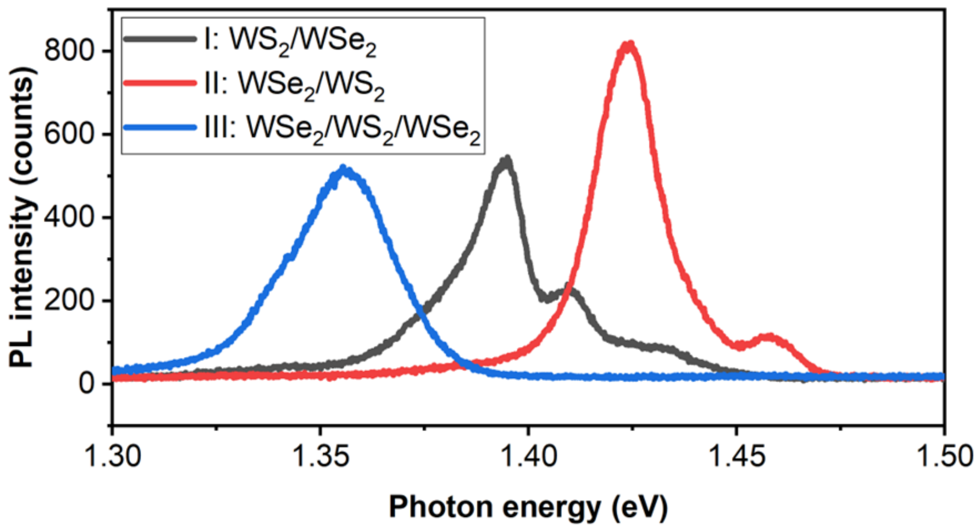

Figure S3. Comparison of the PL spectra from region I, region II and region III at zero gate voltages.

Supplementary section 4: PL spectra from different regions of device D1

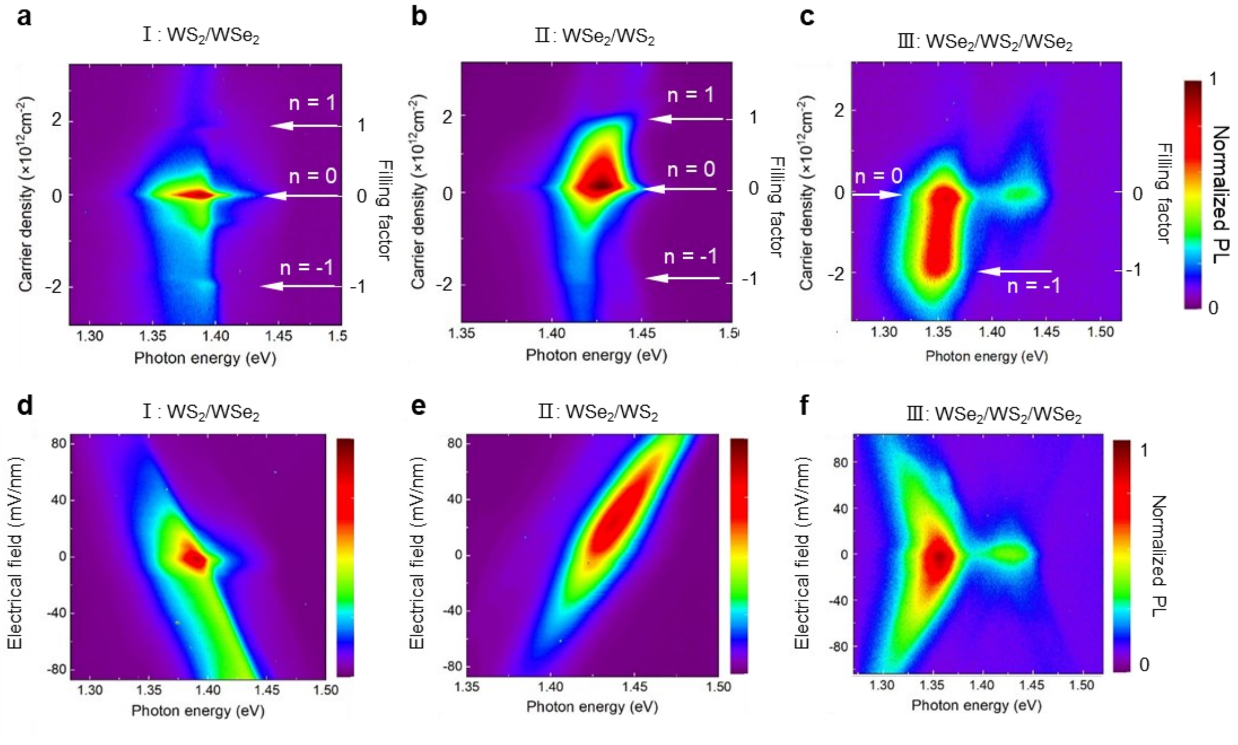

Figure S4. Doping-dependent and electric-field-dependent PL spectra from device D1. (a), (b) and (c) show the doping-dependent PL spectra taken from region I, II and III of device D1, respectively. (d), (e) and (f) show the electric-field-dependent PL spectra taken from region I, II and III of device D1, respectively.

Supplementary section 5: Power dependence of the PL spectra from device D1

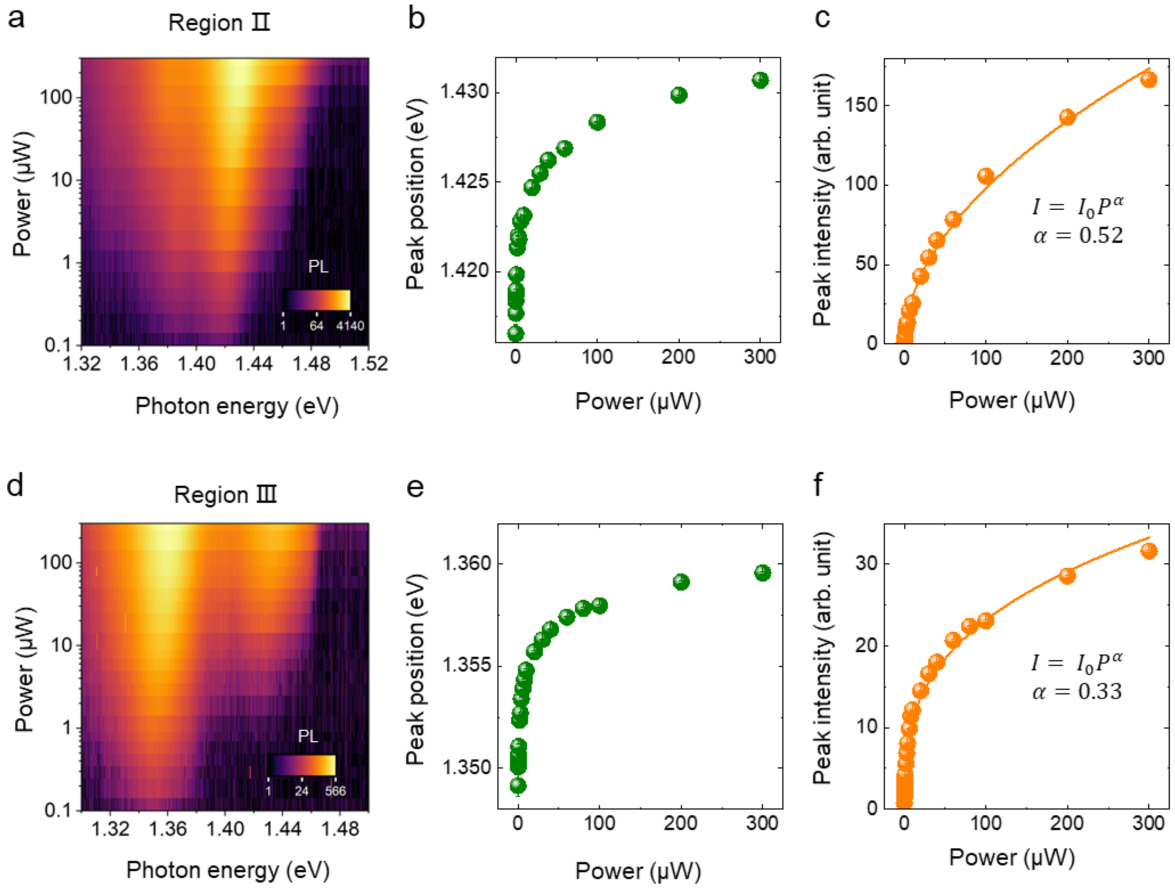

Figure S5. Excitation power dependence of PL from region II and region III (Device D1, shown in the main text). (a) and (d) are PL spectra for different excitation powers from region II and III, respectively. (b) and (e) are PL peak position as a function of excitation power from region II and III. (c) and (f) are integrated PL intensity of excitons as a function of the excitation power from region II and III. The data are taken with the CW excitation with phonon energy centered at 1.959 eV and temperature of 6 K.

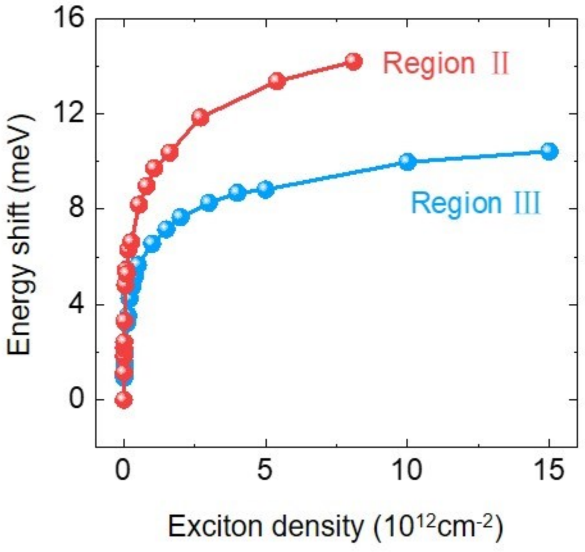

Figure S6. PL peak blueshifts as a function of the exciton density for device D1.

Supplementary section 6: Temperature dependence of the PL spectra from device D1

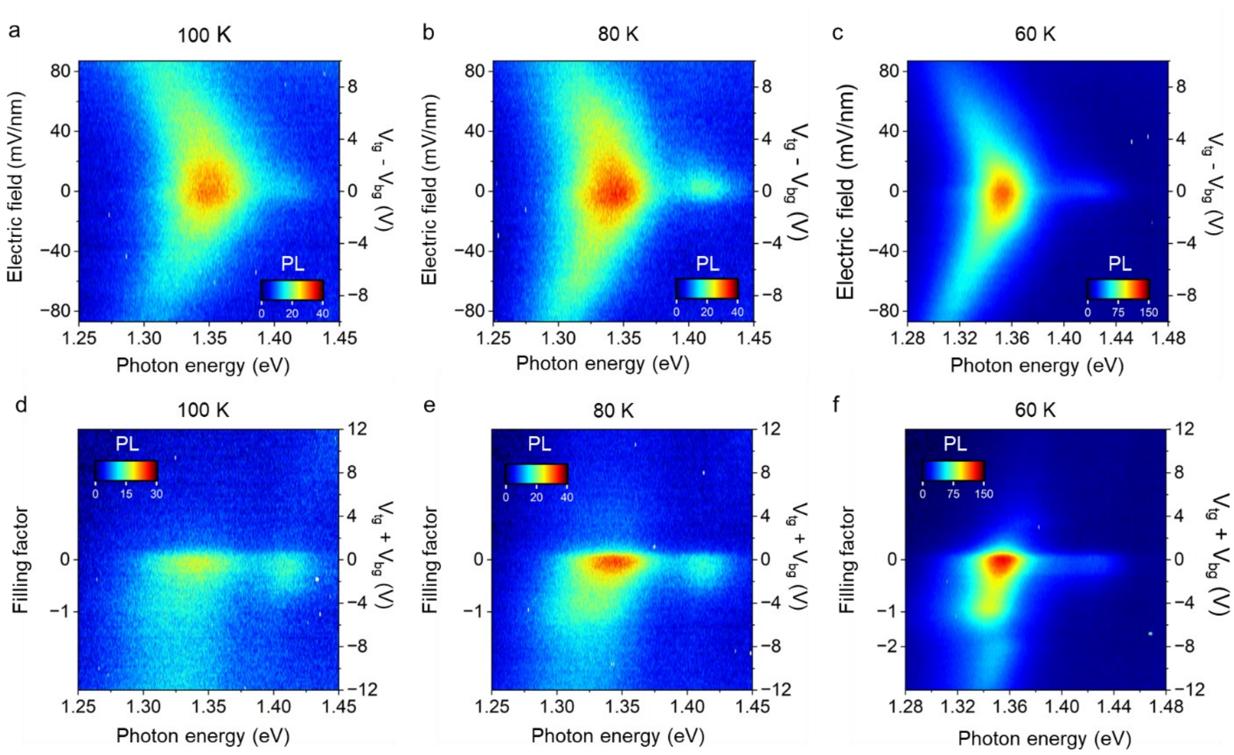

Figure S7. PL spectra of region III measured at different temperatures for device D1. (a)-(c) are the electric-field-dependent PL spectra. (d)-(f) are the doping-dependent PL spectra.

Supplementary section 7: Reflectance contrast measurement and determination of filling factors for device D1

Top-gate-dependent, back-gate-dependent and dual-gate-dependent reflectance contrast spectra with $V_{tg} = V_{bg}$ measured on region I (WS_2/WSe_2) are shown in Figure S8a,b,c. The gate voltages corresponding to n=-1, n=+1 and n=0 can be identified by the abrupt change of resonance positions and intensity in the reflectance contrast spectra, consistent with previous report[1,2]. The gate voltages corresponding to different filling factors are the same in the top-gate-only and bottom-gate-only configuration, indicating an equal thickness of the top BN and the bottom BN. The gate dependence in Figure S8 indicates that the gate voltage between the filling -2, -1, 0 and +1 is ~ 3 V. Using the geometry capacitance model, we estimate the thickness of BN layer to be ~ 31 nm.

The filling factor as a function of gate voltages in Figure S8c is used to calibrate the filling factor of the PL spectra measured from region III, which is done using the same gate configuration. It can be found that the peak position and intensity change in the PL spectra is aligned with the filling factors calibrated with this method.

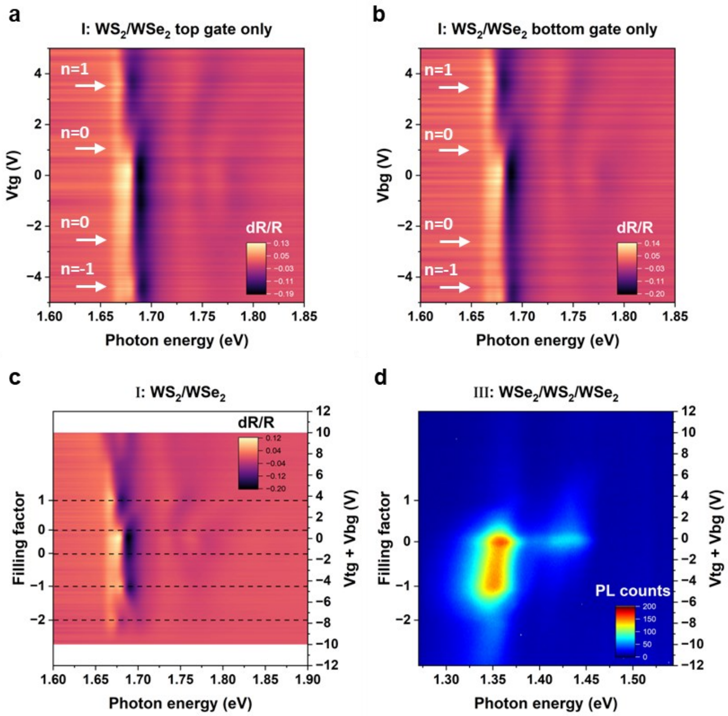

Figure S8. Reflection contrast measurement from region I of device D1 and calibration of filling factors. (a) and (b) show gate-dependent reflection contrast spectra in the region I measured with the top gate and back gate, respectively. (c) shows the reflectance contrast spectra as a function of $V_{tg}+V_{bg}$ while keeping V_{tg} and V_{bg} equal. The dashed lines indicate the filling factors determined from the spectra. (d) shows the gate-dependent PL spectra of device D1. It can be found that at the integer fillings determined in (c), the PL position and intensity exhibit abrupt changes.

Supplementary section 8: Doping-dependent PL spectra at different electric fields and fitting results

Figure S9 shows the PL spectra as functions of the filling factor in the trilayer region (region III) at different positive electric fields. Figure S10 shows the PL spectra as functions of the filling factor from the trilayer region (region III) at different negative electric fields. The PL from the lower energy branch can be fitted with a single Lorentzian peak. The fitted PL peak positions and integrated PL intensities for different electric fields are displayed in Figure S11 and Figure S12, respectively.

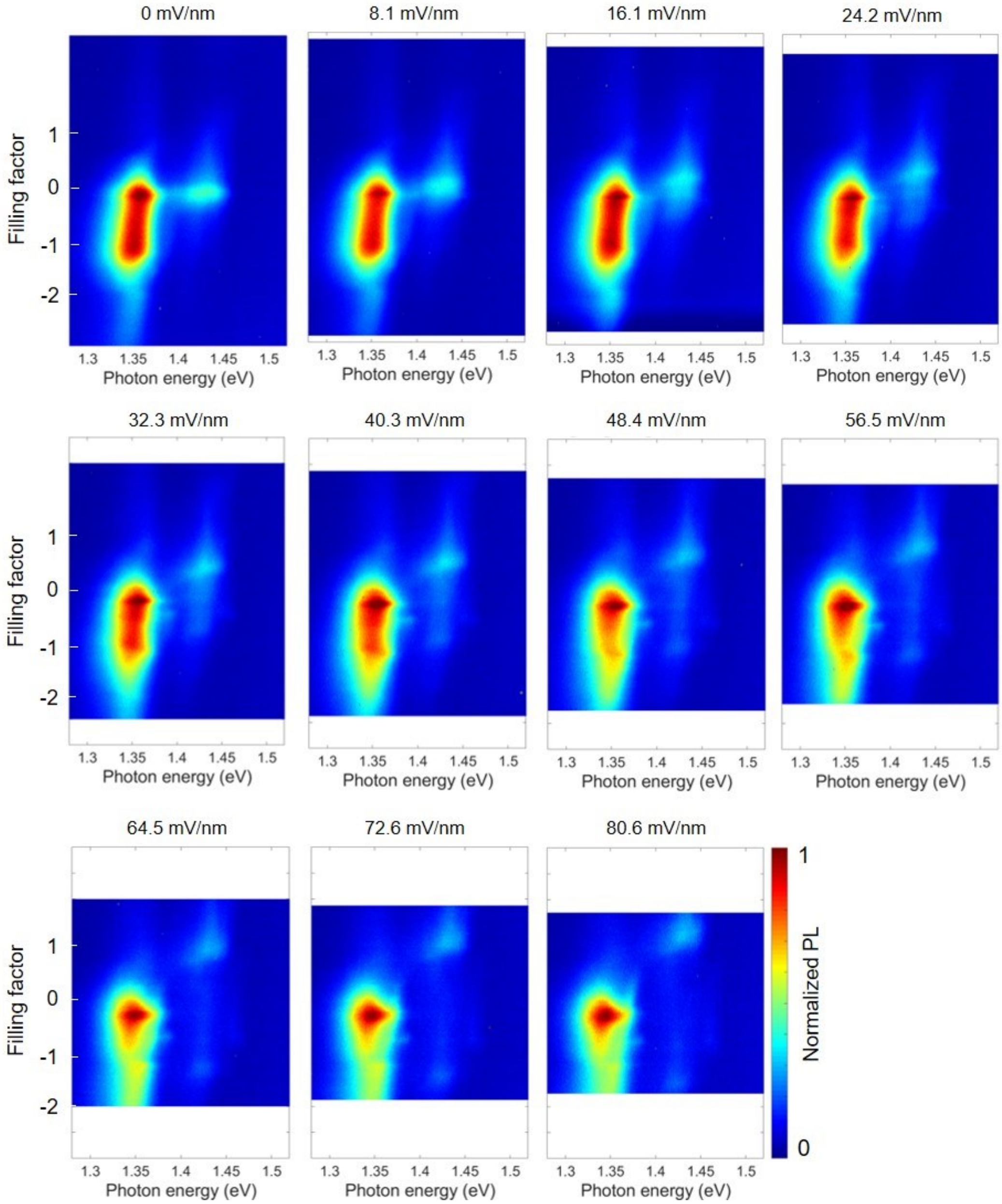

Figure S9. PL spectra as a function of the filling factor from the trilayer region at different positive electric fields.

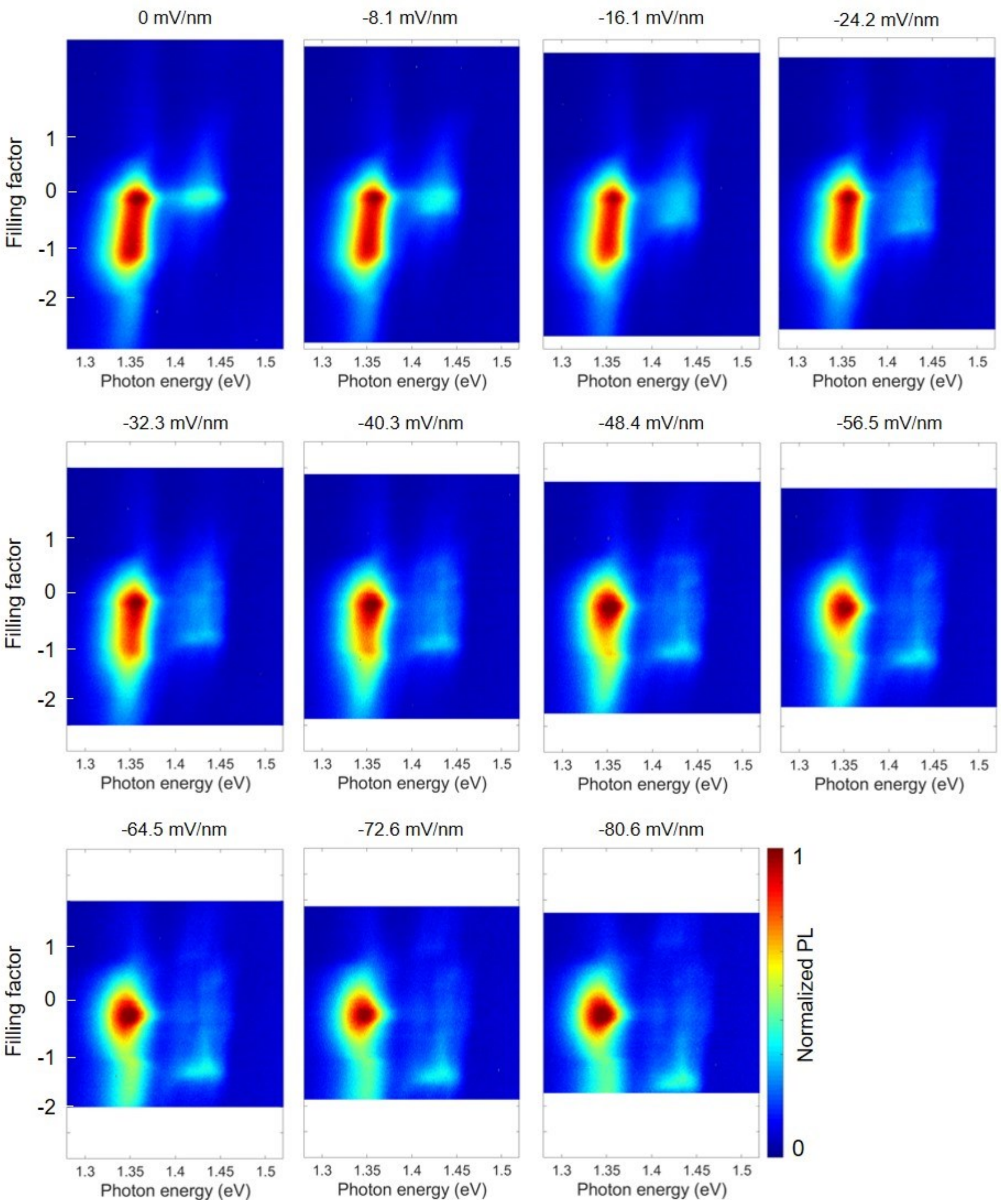

Figure S10. PL spectra as a function of the filling factor from the trilayer region at different negative electric fields.

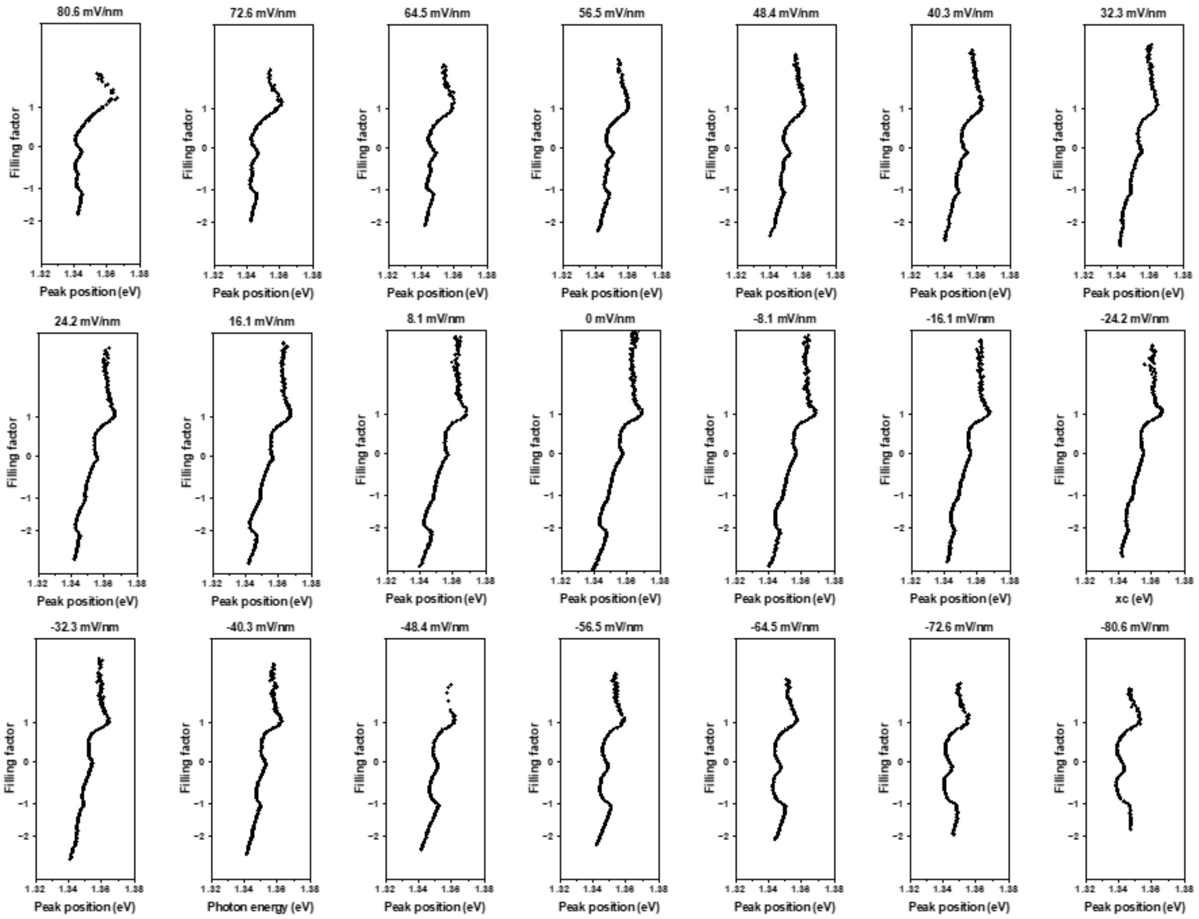

Figure S11. PL Peak positions extracted from Figure S9 and Figure S10 through fitting.

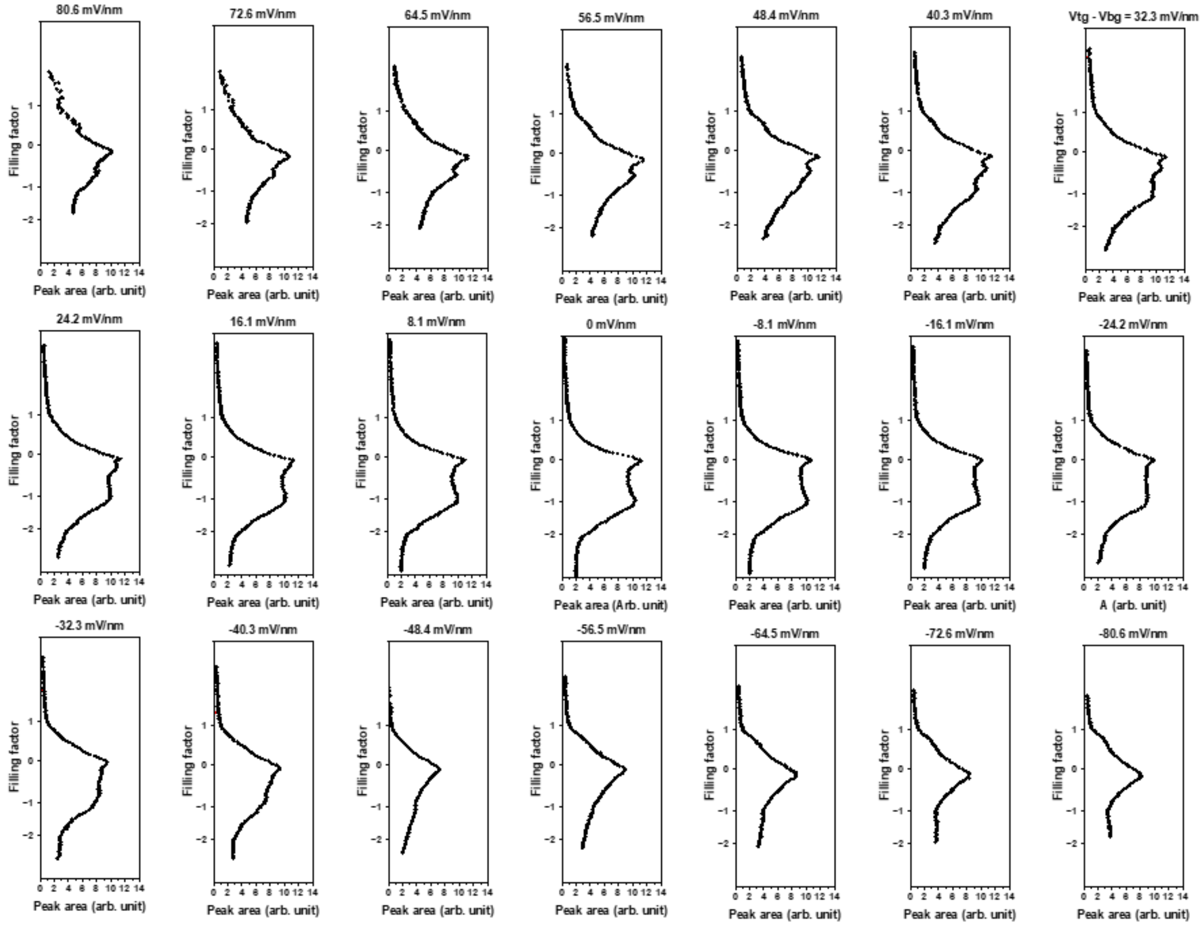

Figure S12. Integrated PL intensity extracted from Figure S9 and Figure S10 through the fitting.

Supplementary section 9: Modeling of quadrupolar excitons

We consider the following two-energy-level Hamiltonian describing the coupling between the two dipolar interlayer excitons:

$$H = \begin{bmatrix} X_1(E) & \Delta \\ \Delta & X_2(E) \end{bmatrix} \tag{9.1}$$

Where X_1(E) and $X_2(E)$ are the dipolar interlayer exciton energies at local electric field E in the WS_2/b-WSe_2 and t-WSe_2/WS_2 heterostructures, respectively, Δ is the coupling between the two interlayer excitons. Solving the eigenvalues of this system, we obtain the expression of the low-energy branch of the hybridized excitons:

$$X_{LE} = \frac{X_1(E) + X_2(E)}{2} - \frac{1}{2}\sqrt{(X_1(E) - X_2(E))^2 + 4\Delta^2} \tag{9.2}$$

The energies of the dipolar excitons can be expressed as:

$$X_1(E) = X_1 - Eed_1 \tag{9.3}$$

$$X_2(E) = X_2 + Eed_2 \tag{9.4}$$

Where X_1 and X_2 are the two dipolar exciton energies at zero electric field.

Eqn. 9.2. is fitted to the extracted peak positions at different electric fields shown in Fig.2d, with X_1, X_2, d_1, d_2 and Δ as fitting parameters. For device D5 shown in Fig.2d, we obtain $X_1 = 1.3696 \pm 0.0002\, eV$, $X_2 = 1.3654 \pm 0.0002\, eV$, $d_1 = 0.659 \pm 0.003\, nm$, $d_2 = 0.740 \pm 0.003\, nm$ and Δ= $12.0 \pm 0.2\, meV$.

Supplementary section 10: Electric field dependent PL spectra in $WSe_2/WS_2/WSe_2$ device D2

Figure S13a shows the electric field dependent PL spectra in another $WSe_2/WS_2/WSe_2$ device. Figure S13b shows the fitting result of the two-level hybridization model. We obtain Δ= 30 ± 9 meV through the fitting shown in Figure S13b.

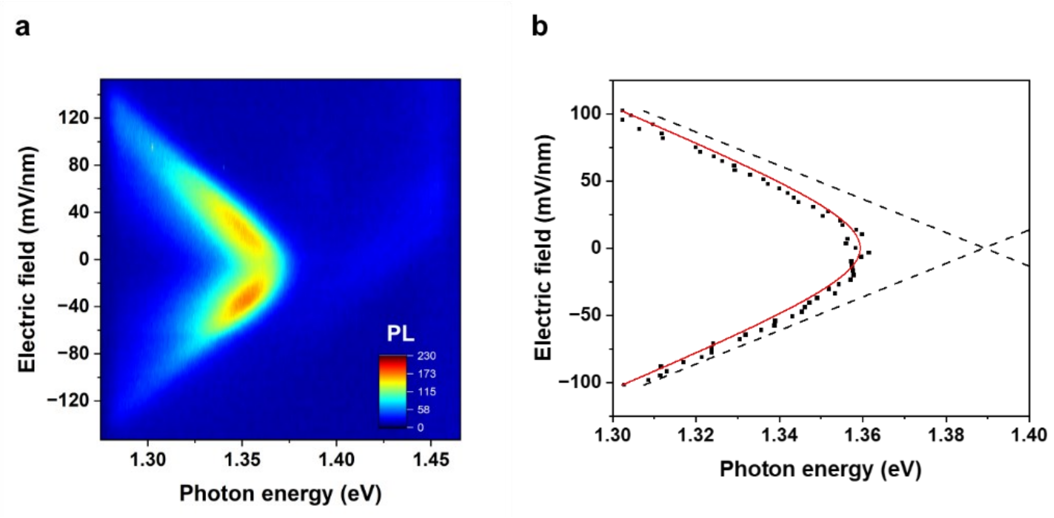

Figure S13. Electric field dependent PL from quadrupolar excitons in another $WSe_2/WS_2/WSe_2$ device (D2). (a) shows the electric field dependent PL spectra from the device D2. (b) shows the fitting result of the two-level hybridization model. The data are taken with a 50 μW CW excitation with photon energy centered at 1.959 eV and a temperature of 9 K.

Supplementary section 11: Calculation of the critical electric field in the TMDC heterostructure at n=-1 and n=-2

We define E_t, E_b, and E_h as the electric field inside top BN, bottom BN and TMDC heterostructure, d_t, d_b and d_h as the thickness of top BN, bottom BN and the TMDC heterostructure. Here in our case d_t = d_b = $d \approx$ 31 nm. The dielectric constants of BN and TMDC are denoted as ε_{BN} and ε_{TMDC}, respectively. Here we consider the electric field inside the TMDC heterostructure in three special cases: (1) the heterostructure is charge-neural; (2) n = -1 and all the holes reside in the b-WSe_2 layer; and (3) n=-2 and holes are distributed equally in the top and bottom WSe_2. The corresponding charge and electric field distribution are shown in Figure S14 (a), (b) and (c), respectively.

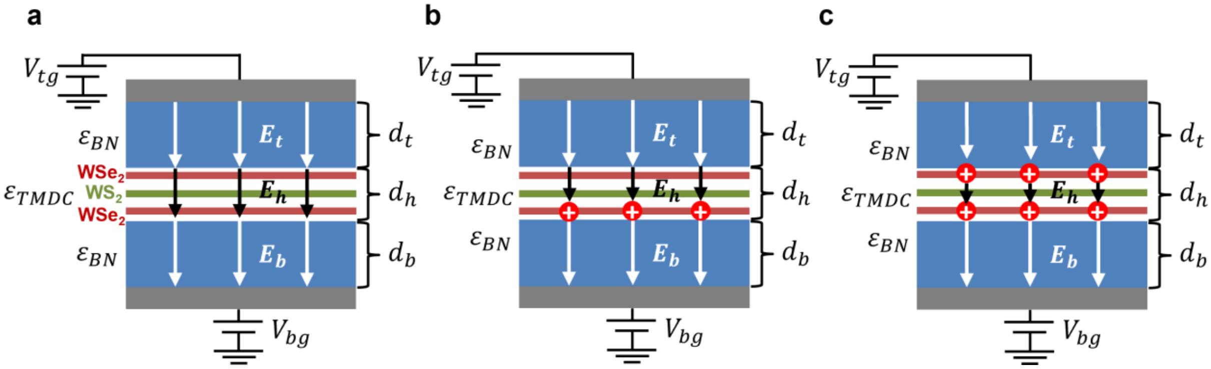

Figure S14. Schematics of the charge distribution in the trilayer region at n=0 (a), n=-1(b), and n=-2 (c).

Case 1: The heterostructure is in charge neutral region (n=0):

When the sample is insulating, there's a large potential drop on the quantum capacitance between the device and the ground[3,4]. Therefore, the heterostructure can be seen as floated. Considering the boundary conditions at the boundaries of dielectrics, we obtain the following equations:

$$\varepsilon_{BN}E_t = \varepsilon_{TMDC}E_h \tag{11.1}$$

$$\varepsilon_{BN}E_b = \varepsilon_{TMDC}E_h \tag{11.2}$$

Considering the potential drop between the top gate and the bottom gate, we have:

$$E_t d_t + E_h d_h + E_b d_b \approx E_t d_t + E_b d_b = V_{tg} - V_{bg} \tag{11.3}$$

Here we neglected the potential drop in the TMDC heterostructure because its thickness is significantly smaller than the thickness of the BN layers.

From the above equations, we obtained the electric field inside the heterostructure:

$$E_h = \frac{\varepsilon_{BN}}{\varepsilon_{TMDC}} \frac{V_{tg} - V_{bg}}{2d} \tag{11.4}$$

Case 2: n = -1

Then we consider the threshold electric field to make one of the bilayers at n = -1 and the other at n = 0. In addition to the applied field between top gate and bottom gate, we need to consider the electric field generated by correlated holes. Let us consider the case where $V_{tg} > V_{bg}$ and holes only occupy the bottom WSe_2 layer. The boundary conditions become:

$$\varepsilon_{BN} E_t = \varepsilon_{TMDC} E_h \tag{11.5}$$

$$\varepsilon_{BN} E_b - \varepsilon_{TMDC} E_h = n_0 e \tag{11.6}$$

where $n_0 = 2.1 \times 10^{12} cm^{-2}$ is hole density corresponding to one hole per unit cell and e is the elementary charge. Therefore, we obtain:

$$E_h = \frac{\varepsilon_{BN}}{\varepsilon_{TMDC}} \frac{V_{tg} - V_{bg}}{2d} - \frac{n_0 e}{2\varepsilon_{TMDC}} = \frac{\varepsilon_{BN}}{\varepsilon_{TMDC}} E_{ext} - \frac{n_0 e}{2\varepsilon_{TMDC}} \tag{11.7}$$

where E_{ext} is the external electric field defined in the Method Section of the main text. In our calculation we use dielectric constant values $\varepsilon_{BN} = 3.9\varepsilon_0$ and $\varepsilon_{TMDC} = 7.2\varepsilon_0$ [4]. Considering the critical external field of 44 mV/nm obtained from Fig. 4b, we obtain $E_h = -2.5\, mV/nm \approx 0.00 V/nm$

Case 3: n=-2

At n=-2, there are symmetric amounts of holes occupying both the top WSe_2 layer and the bottom WSe_2 layer. The boundary conditions are:

$$\varepsilon_{TMDC} E_h - \varepsilon_{BN} E_t = n_0 e \tag{11.8}$$

$$\varepsilon_{BN} E_b - \varepsilon_{TMDC} E_h = n_0 e \tag{11.9}$$

Hence, we obtain:

$$E_h = \frac{\varepsilon_{BN}}{\varepsilon_{TMDC}} \frac{V_{tg} - V_{bg}}{2d} = \frac{\varepsilon_{BN}}{\varepsilon_{TMDC}} E_{ext} \tag{11.10}$$

From Fig. 4c, the critical external electric field to make the peak shift at n=-2 disappear is ~ 32.2 mV/nm. Therefore, we have $E_h = 17.4\, mV/nm$ in this case. According to the interlayer distance of about 1.2 nm we extracted from the dipolar excitons fitting

(Supplementary Section 9), we obtain the energy difference between the two WSe_2 layers induced by the electrostatic potential at the critical external electric field to be about 20 meV.

Supplementary section 12: Electric field dependence and doping dependence of PL spectra measured from device D3

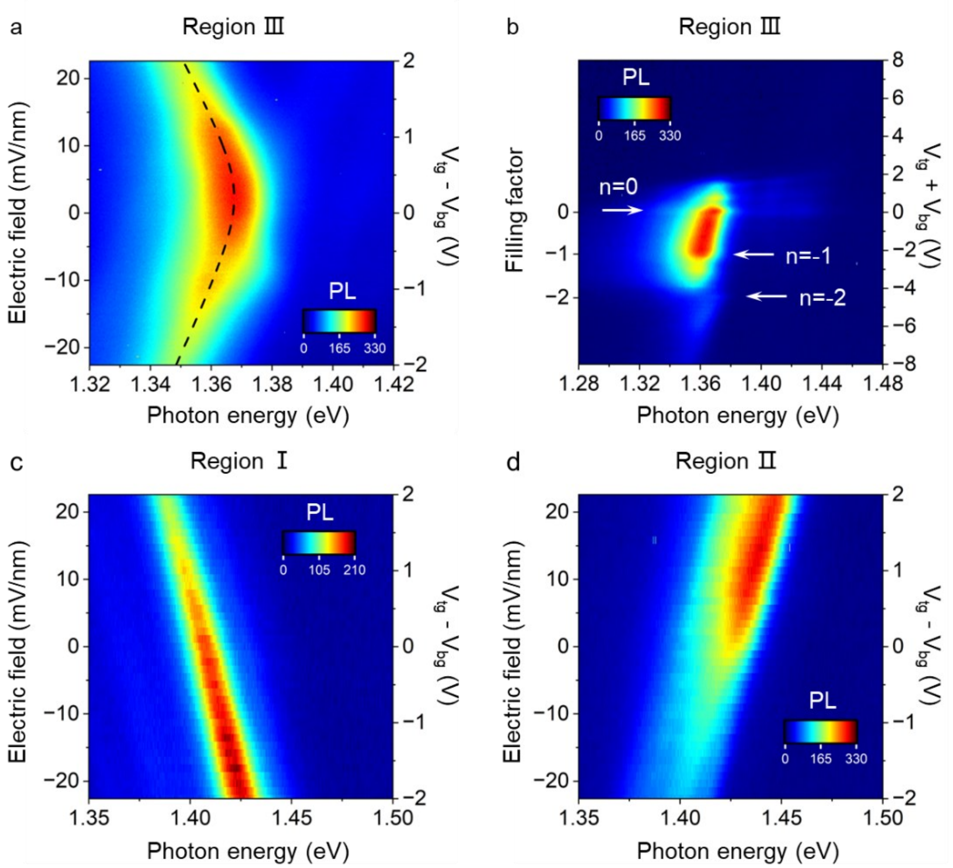

Figure S15. Electric field dependent and doping dependent PL spectra of another device D3. The data are taken with a 10 μW CW excitation with phonon energy centered at 1.959 eV and a temperature of 6 K.

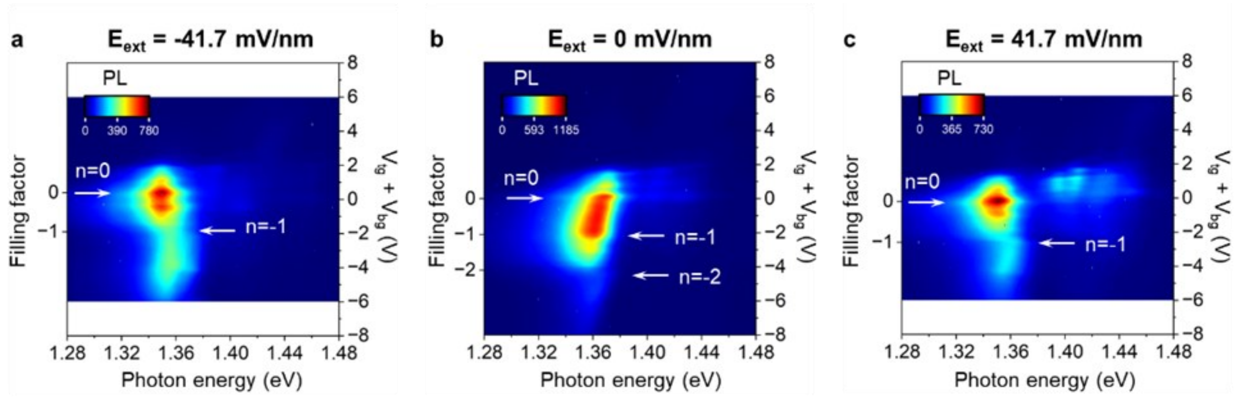

Figure S16. Doping-dependent PL spectra from device D3 at different electric fields. (a), (b) and (c) show the PL spectra taken from region III of device D3 with an out-of-plane external electric field of -41.7 mV/nm, 0 mV/nm and 41.7 mV/nm, respectively. The PL spectra are taken at 6K, with 10 μW 1.959 CW excitation.

Supplementary section 13: Power dependence of PL spectra of device D3

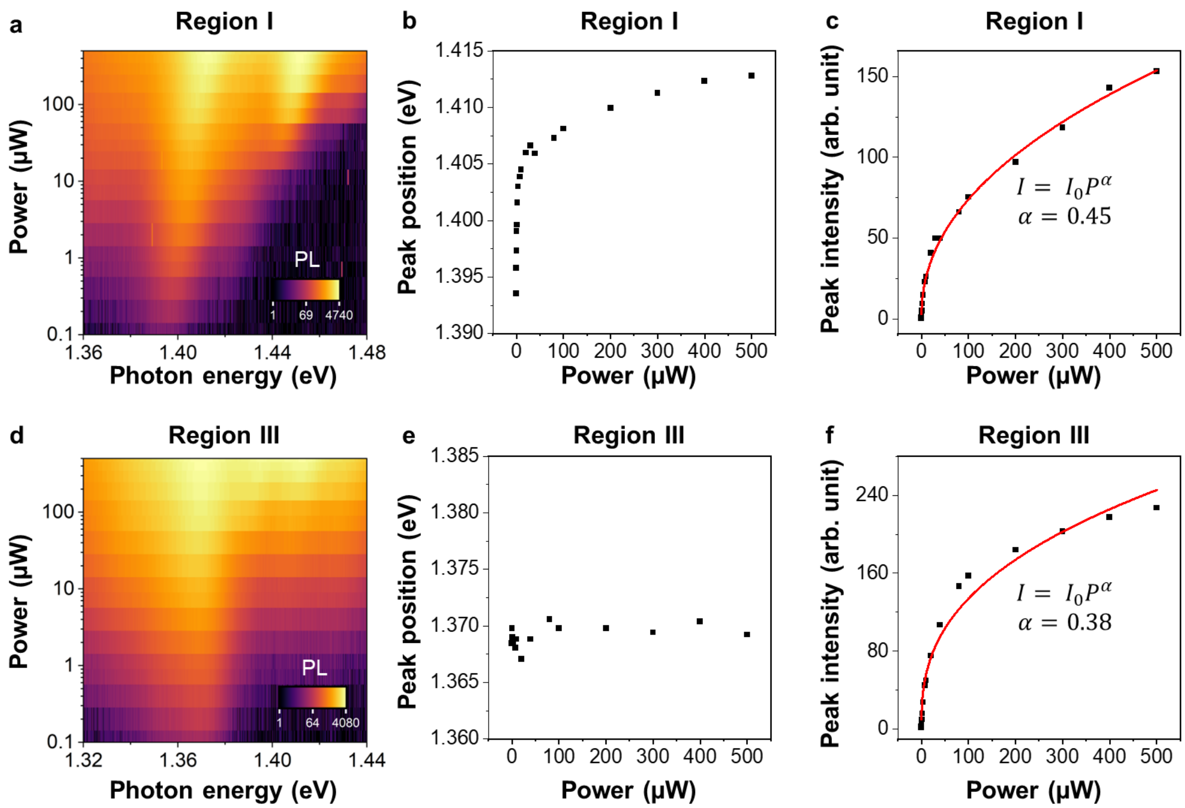

Figure S17. Excitation power dependence of PL spectra for region I and III of device D3.

Supplementary section 14: Fitting to the two-level hybridization model for device D1 and D3

Here we fit the electric field dependence of quadrupolar exciton PL peak positions from device D1 and D3 to the model described in supplementary section 9. For device D1, we obtain $\Delta = 9 \pm 2$ meV, $X_1(0) = 1.368 \pm 0.001$ eV and $X_2(0) = 1.365 \pm 0.001$ eV. For device D3, the fitting yields $\Delta = 9 \pm 1$ meV, $X_1(0) = 1.380 \pm 0.001$ eV and $X_2(0) = 1.373 \pm 0.001$ eV.

Although the two dipolar excitons are of slightly different energies in device D3, they still form quadrupolar excitons due to the electric field tunability (schematically shown in Fig. S19). We note that the schematic shown in Fig. S19 also offers a way to experimentally determine whether the two dipolar excitons are of similar energies in the trilayer region: whether the quadrupolar excitons energy maximum, indicated by the arrow in Fig. S19, is at zero electric field or finite electric field. It is thus clear that the two dipolar excitons are of similar energies in device D1 (Fig. S18a) but slightly different in device D3 (Fig. S18b), consistent with the fitting results.

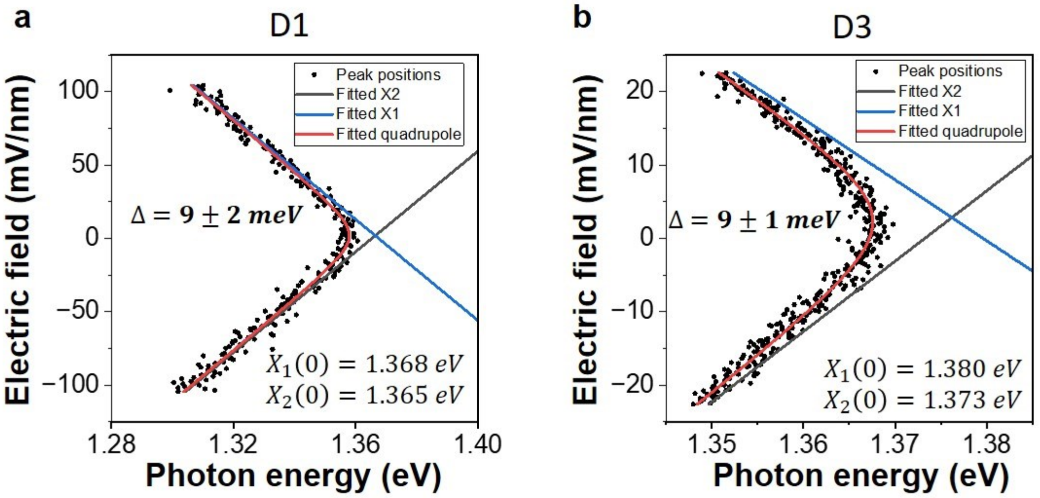

Figure S18. Fitted dipolar exciton and quadrupolar exciton energies obtained by setting the dipole moments and the peak positions at zero electric field of the two dipolar excitons as independent parameters from device D1 and D3.

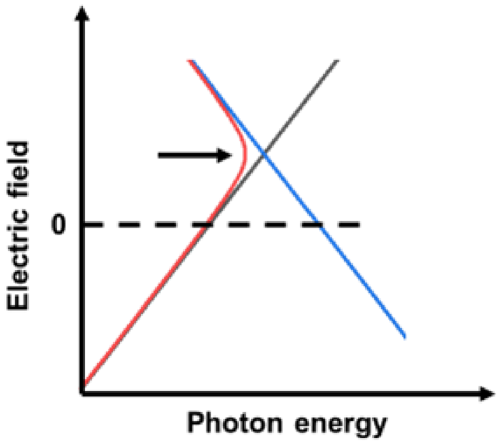

Figure S19. A schematic showing the hybridization of dipolar excitons with different energies at zero electric field.

We have observed the signs of interlayer quadrupolar exciton PL in 19 devices, and 9 of them can be well fitted with the quadratic electric field dependence. Among them, the interlayer Mott insulator behavior presented in the main text is observed in 6 devices. We suspect that the observation of the correlated states is more demanding on the relative angle alignment between the top and bottom WSe_2 layer, which is needed to ensure the same moiré supercell for the top and bottom moiré bilayer.

Supplementary section 15: PL spectra of device D1 measured with 1.698 eV laser excitation

Here we show the PL spectra of device D1 with the exciton photon energy of 1.698 eV, which is in resonance with the moiré A exciton of WSe_2. The spectra are similar to what we show in the main text and supplementary section 4. Both excitation photon energy of 1.959 eV and 1.698 eV are much larger than the interlayer exciton resonance of ~1.4 eV.

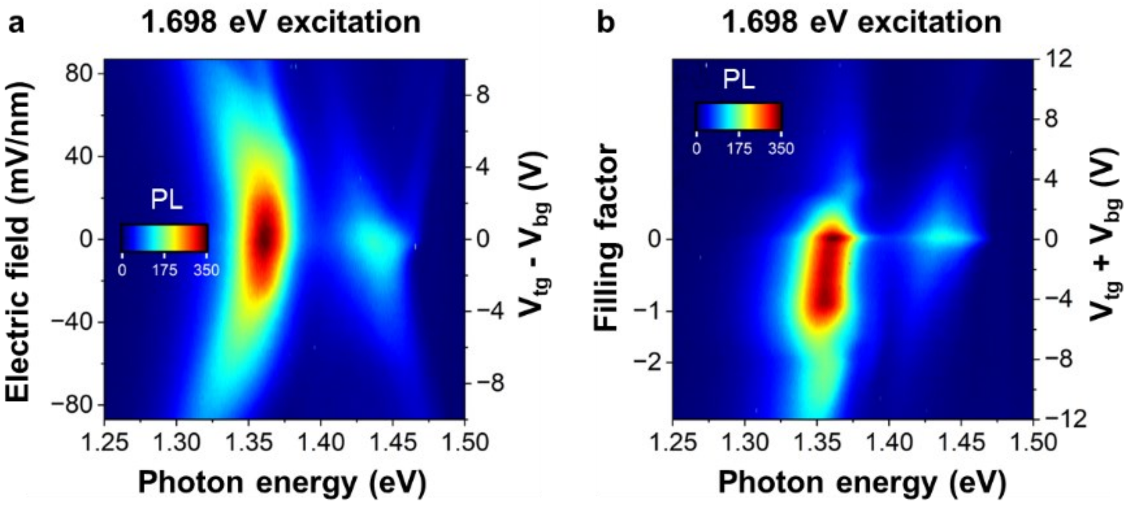

Figure S20. Electric field dependence and doping dependence of the PL spectra from region III of device D1. CW laser with the phonon energy centered at 1.698 eV, in resonance with the moiré A exciton of WSe_2, is used as the excitation. The excitation power is 200 μW, and the data are taken at a temperature of 6 K.

Supplementary section 16: Estimation of exciton density

Here we derive the exciton density as a function of excitation power following the method described in ref.5. We consider the reflection and absorption of each layer of material. The effective power density at the heterobilayer is expressed as:

$$P_{eff} = P(1 - R_G - A_G)(1 - R_{BN})(1 - R_{TMDC}) \qquad (16.1)$$

Where A_G and A_{TMDC} denote the absorptance of the top graphite and the top BN, respectively. R_G, R_{BN} and R_{TMDC} denote the reflectance of the top graphite, top BN and the heterobilayer. P is the power density at the surface of the sample, which can be calculated using the excitation power and the beam spot size d = 2 μm. For few-layer graphene top gate, the absorptance A_G is negligible. At each dielectric interface, the reflectance R can be calculated by:

$$\mathrm{R}(\lambda) = \left|\frac{n_R(\lambda) - n_T(\lambda)}{n_R(\lambda) + n_T(\lambda)}\right|^2 \qquad (16.2)$$

Where $n_R(\lambda)$ and $n_T(\lambda)$ are the real refractive indexes at the reflectance side and the transmittance side, respectively.

Using the refractive index values adapted from previous works[6-9], we estimate $P_{eff} = 0.64P$ for 1.96 eV excitation.

We estimate the proportion of the power absorbed by the hetero-trilayer using the values in ref.6, which is 0% for WS_2 and 2% for WSe_2 at 1.96 eV. For region III, assuming 4% absorption and unitary conversion efficiency, we obtain the exciton generation rate $\mathrm{g} = 2.6 \times 10^{18}\ cm^{-2} \cdot s^{-1}$ for 1 μW 1.96 eV excitation. Using a measured exciton lifetime around 19 ns, the steady-state exciton density can be estimated by $d_{ex} = g \cdot \tau$ which is $5.0 \times 10^{10} cm^{-2}$ for 1 μW excitation. With 200 μW 1.96 eV excitation, the steady-state exciton density is $1.0 \times 10^{13} cm^{-2}$for 200 μW 1.96 eV excitation and $2.5 \times 10^{12} cm^{-2}$ for 50 μW 1.96 eV. Similarly, for region II, 1 μW excitation power corresponds to an exciton density of $2.7 \times 10^{10} cm^{-2}$.

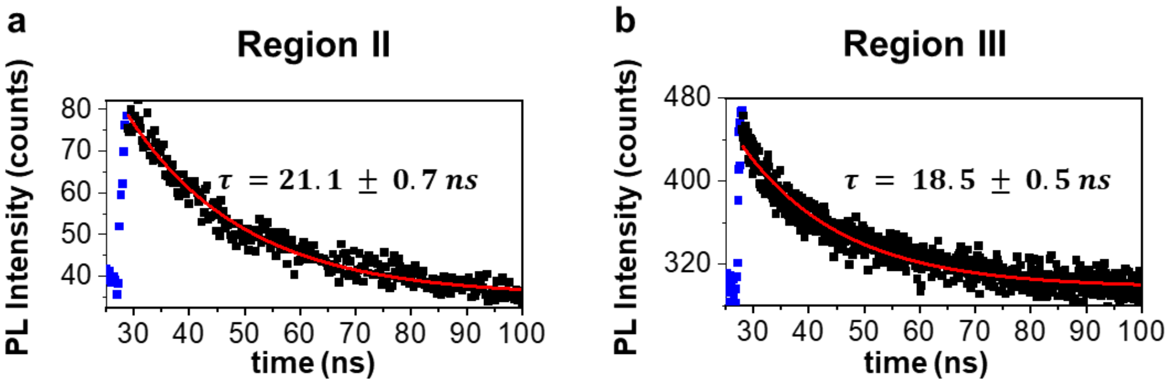

Figure S21. Time-resolved photoluminescence (TRPL) measured from region II and region III. (a) and (b) are the TRPL data taken from region II and region III of device D1, respectively. The red lines indicate fitting results using an exponential decay function $I(t) = I_0 e^{(t-t_0)/\tau}$. The TRPL measurements were performed using a pulsed laser centered at 1.771 eV with a repetition rate of 10 MHz. The excitation power is 400 μW.

Supplementary section 17: Discussion of the energy scales related to the interlayer Mott state

The n=-2 state in Fig. 3 is the case where the top and bottom moiré superlattices are half-filled by holes (-1 for the top WSe_2/WS_2 and -1 for the bottom WS_2/WSe_2 moiré superlattice). Therefore, the bandgap will be the smaller onsite repulsion energy (U) of the top and bottom Mott insulator minus the energy $\Delta^{\pm}$. In Fig. S22, we assume the same U for both valence bands for simplicity. We believe that this U could be more than 50 meV (manuscript under preparation and a recent work on arXiv[10]).

The n=-1 state in Fig. 3 is the newly discovered hybridized Mott insulator state. It can be understood schematically as shown in Fig. S22. The hybridization of the flat valance band gives to the bonding state (share the origin of the quadrupolar excitons) and antibonding state, separated by the energy gap $\Delta^{\pm}$. For the n=-1 state, the bonding state will be half filled as the LHB, while the UHB of the bonding state will be at the energy U higher. As we extracted Δ to be 9 meV (SI Section 14) for device D1, which corresponding to a $\Delta^{\pm}$ of 18 meV, the UHB will be higher than the antibonding state, so that the next added electron will occupy the antibonding state, leading to the bandgap of $\Delta^{\pm}$. As $\Delta^{\pm}$ is smaller than U-$\Delta^{\pm}$, we would expect the shift at n=-1 to be smaller than that at the n=-2.

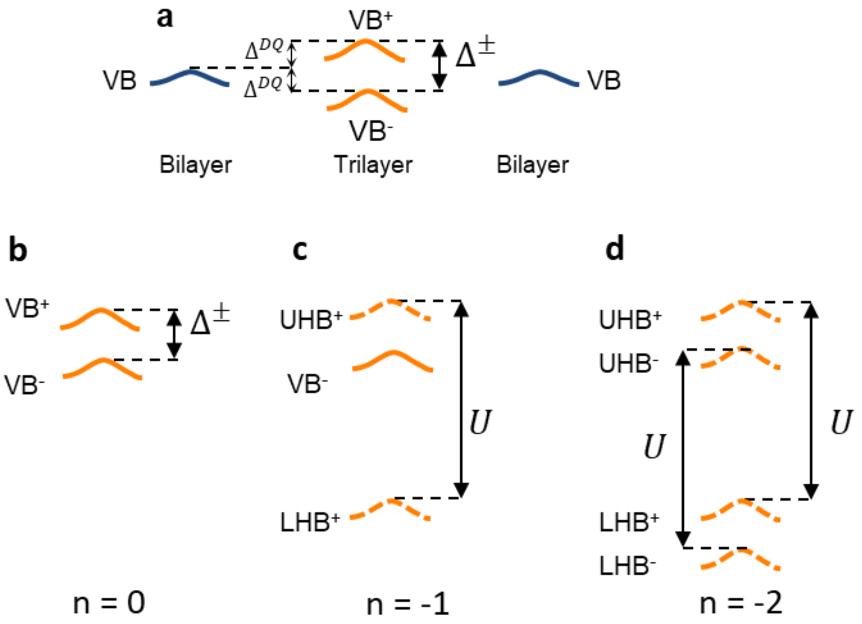

Figure S22. Schematics showing the interlayer hybridized Mott insulating state. (a) shows the valence band hybridization process in $WSe_2/WS_2/WSe_2$ trilayers. (b), (c) and (d) show the schematics of the bandstructure in the trilayer region at hole filling of n=0, n=-1 and n=-2, respectively

Supplementary section 18: Discussion of the nature of the high-energy peak from device D1

The high-energy PL peaks in Fig. 3 and Fig. S4 occur at high excitation power and are not universal among all the devices we have studied. It could be attributed to other exciton modes at higher energy, including possible quadrupolar exciton modes from hybridization of higher energy dipolar excitons. We do not believe that it is from the high energy branch (antisymmetric mode associated with the symmetric quadrupolar exciton mode discussed in the main text) quadrupolar exciton (because of the large energy separation from the ground state quadrupolar exciton. It is likely that the simplified model did not consider moiré effects or possible complications from other dipolar excitons (such as spin-singlet exciton[11]). As the PL spectra are most sensitive to the ground state, we focus on the symmetric quadrupolar exciton branch (lower energy branch) in this work and leave the investigation of the high energy mode(s) to future exploration.

Supplementary section 19: PL spectra from a $WS_2/WSe_2/WS_2$ heterostructure

Fig.S23 shows the doping-dependent and electric-field-dependent of PL spectra measured from a $WS_2/WSe_2/WS_2$ device (D4).

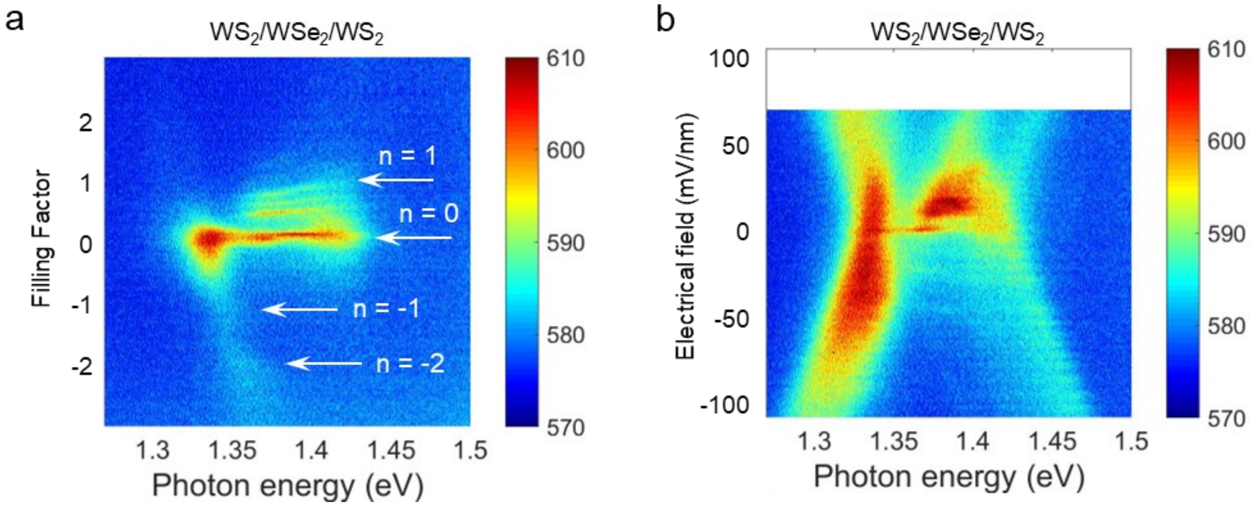

Fig.S23. PL spectra from a $WS_2/WSe_2/WS_2$ heterostructure. (a) and (b) show the doping dependence and the electric field dependence of PL spectra from $WS_2/WSe_2/WS_2$ device D4.

References

1. Regan, E. C. *et al.* Mott and generalized Wigner crystal states in WSe_2/WS_2 moiré superlattices. *Nature* **579**, 359–363 (2020).

2. Tang, Y. *et al.* Simulation of Hubbard model physics in WSe_2/WS_2 moiré superlattices. *Nature* **579**, 353–358 (2020).

3. Zhang, Y. *et al.* Direct observation of a widely tunable bandgap in bilayer graphene. *Nature* **459**, 820–823 (2009).

4. Jauregui, L. A. *et al.* Electrical control of interlayer exciton dynamics in atomically thin heterostructures. *Science* **366**, 870–875 (2019).

5 Bai, Y., *et al.* Evidence for exciton crystals in a 2D semiconductor heterotrilayer. arXiv:2207.09601 (2022).

6 Li, Y. *et al.* Measurement of the optical dielectric function of monolayer transition-metal dichalcogenides: MoS_2, $MoSe_2$, WS_2, and WSe_2. *Physical Review B* **90**, 205422 (2014).

7. Lee, S.‐Y. *et al.* Refractive index dispersion of hexagonal boron nitride in the visible and near‐infrared. *Phys. Status Solidi B* **256**, 1800417 (2019).

8. Wang, X. *et al.* Strong anomalous optical dispersion of graphene: complex refractive index measured by picometrology. *Optics Express* **16**, 22105-22112 (2008).

9. Hsu, C. *et al.* Thickness‐dependent refractive index of 1L, 2L, and 3L MoS_2, $MoSe_2$, WS_2, and WSe_2. *Advanced optical materials* **7**, 1900239 (2019).

10. Li, H. *et al.* Mapping Charge Excitations in Generalized Wigner Crystals. arXiv:2209.12830v2

11. Wang, T. *et al.* Giant Valley-Zeeman Splitting from Spin-Singlet and Spin-Triplet Interlayer Excitons in $WSe_2/MoSe_2$ Heterostructure. *Nano Lett.* **20**, 694–700 (2020).
